# Dry Needling and Acupuncture for Scars—A Systematic Review

**DOI:** 10.3390/jcm13143994

**Published:** 2024-07-09

**Authors:** Robert Trybulski, Adam Kawczyński, Jarosław Muracki, Nicola Lovecchio, Adrian Kużdżał

**Affiliations:** 1 Provita Żory Medical Center, 44-240 Żory, Poland; rtrybulski.provita@gmail.com; 2 Medical Department Wojciech Korfanty, Upper Silesian Academy in Katowice, 40-659 Katowice, Poland; 3 Department of Paralympic Sport, Wroclaw University of Health and Sport Sciences, 51-612 Wrocław, Poland; 4 Institute of Physical Culture Sciences, Department of Physical Culture and Health, University of Szczecin, 70-453 Szczecin, Poland; 5 Department of Human and Social Sciences, University of Bergamo, 24129 Bergamo, Italy; 6 Institute of Health Sciences, College of Medical Sciences, University of Rzeszów, 35-310 Rzeszów, Poland

**Keywords:** percutaneous needle therapy, interventional physiotherapy skin, keloid

## Abstract

**Objectives**: This research aims to synthesize existing data on the evidence gap in scar treatment and evaluate the effectiveness of acupuncture and dry needling in treating scars and related symptoms. **Methods**: The article adhered to the PRISMA 2020 statement for recommended reporting elements in systematic reviews. The inclusion criteria followed the PICO methodology. The literature search was conducted using databases including PubMed, Cochrane Library, Semantic Scholar, Europe PubMed Central, and Google Scholar. Studies on acupuncture and dry needling for scar treatment were included. Because of the diversity of the studies’ results and methodologies, a systematic review was conducted to organize and describe the findings without attempting a numerical synthesis. **Results**: Nineteen studies relevant to the article’s theme were identified, with eleven selected for detailed review. The studies included two case reports on dry needling, one case series on dry needling, five case reports on acupuncture, two randomized controlled trials on acupuncture, and one case report on Fu’s subcutaneous needling. A quality assessment was conducted using the JBI CAT and PEDro scales. Four case reports scored 7 points, one case scored 8 points, three cases were rated 6 points or lower, the case series was rated 6 points, and the randomized controlled trials scored 8 and 5 points. Most studies demonstrated a desired therapeutic effect in scar treatment with acupuncture and dry needling, but the level of evidence varied across studies. The analysis does not conclusively support the use of acupuncture and dry needling to improve scar conditions. **Conclusions**: Although dry-needling and acupuncture techniques are popular in physiotherapy, adequate scientific evidence is currently not available to support their effectiveness in scar treatment. There are gaps in the research methodology, a lack of randomized trials, and significant heterogeneity in the assessment of effects.

## 1. Introduction

Scars can cause both physical and emotional difficulties for patients. People seek effective but less invasive methods to address issues such as scars. Acupuncture (ACU) and dry needling (DN) are highly debated methods, yet they are widely used, and there has been a recent increase in interest in learning about these approaches. Exploring alternative treatment methods may be beneficial for those who prefer not to use traditional scar treatment methods. Currently, there is a lack of sufficient research or empirical data confirming the effectiveness of dry needling and acupuncture in scar treatment. There is the need for a systematic review of existing scientific publications and clinical trials to determine the presence of specific evidence.

Scar is a fibrous tissue that forms as a result of damage to the skin or other tissues during the healing process rather than through regeneration. Scars are natural outcomes of wound healing and can be keloid or hypertrophic. Approximately 48.5% of adults have at least one scar, with 12.3% of men and 10.7% of women experiencing painful scars [1], impacting their appearance and physical well-being. According to some studies, the use of acupuncture may improve the condition of scars and alleviate associated symptoms [2,3].

Scars can cause pain, itching, a deterioration of tissue elasticity, restricted mobility due to contractures, and social distress [4]. Keloids are excesses of dense fibrous tissue that extend beyond the original wound edge, and they have a tendency to recur after therapy. Hypertrophic scars are erythematous, itching formations that remain within the boundaries of the initial injury. They can be categorized as linear (resulting from surgery or trauma) or widespread (due to extensive burns or soft-tissue infections). The exact mechanism of hypertrophic and keloid scar formation is not fully understood, but one theory involves a disruption in one of the phases of healing (inflammation, proliferation, or maturation). Fibroblasts, vascular endothelial cells, and numerous inflammatory cytokines are involved in scar tissue formation [5]. Fibroblasts produce collagen, which can directly impact the wound microenvironment and serve as a framework for cell attachment [6]. In cases in which the balance between the anabolic and catabolic stages of healing is disrupted, collagen production outweighs the breakdown, causing the scar to grow in all directions [7].

**Figure 1:**
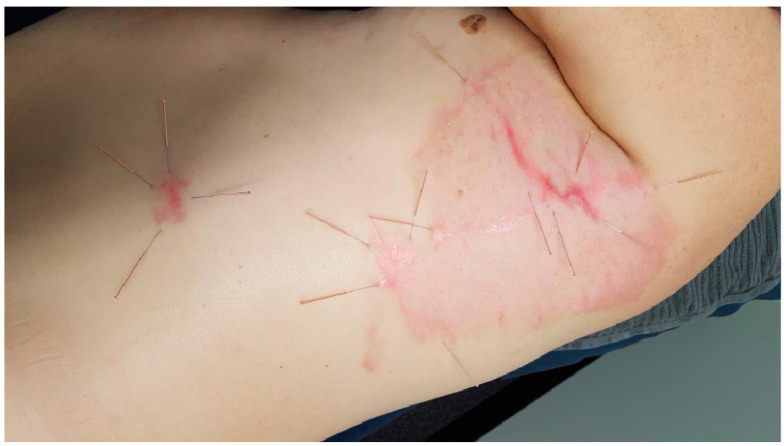
Application of acupuncture in scar treatment. Source: Tuckey et al. (2022) [3].

Acupuncture is a treatment system rooted in the traditional Chinese medicine (TCM) paradigm. On the basis of the stimulation of specific points on the body, it employs thin metal needles or pressure to activate these points, which are located along 12 bilateral meridians [8]. Manual acupuncture involves various needle movements, such as lifting, thrusting, and rotating. The potential pain relief mechanism arises through the impact on the limbic system and the activation of afferent fibers. This stimulates the spinal cord, leading to the synthesis of dynorphin, enkephalin, serotonin, and norepinephrine. This process inhibits the transmission of pain in both pre- and postsynaptic pathways in the spinal–thalamic tract [9]. Acupuncture is a safe treatment method, but it may have side effects such as bruising and minimal bleeding. Figure 1 depicts an example of acupuncture’s application in scar treatment.

**Figure 2:**
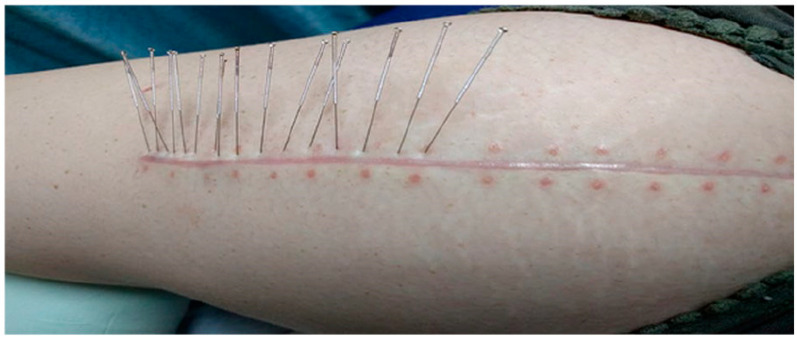
Application of dry needling in scar treatment. Source: Rozenfeld et al. (2020) [16].

Dry needling is a therapeutic technique that involves the insertion of thin, solid needles into myofascial trigger points or tight bands of skeletal muscle [10]. As per the American Physical Therapy Association, DN is described as a specialized technique involving the use of a thin needle to penetrate the skin and activate underlying muscles and connective tissues, aiming to address neuromuscular and movement impairments [11]. Unlike traditional acupuncture, dry-needling targets myofascial trigger points to release tension and alleviate pain [12]. The needles are typically the same as those used in acupuncture, but the approach focuses on addressing muscular issues [13]. In sports, dry needling is often employed to enhance athletic performance, treat musculoskeletal injuries, and manage pain [14]. Dry-needling targets specific muscle knots or trigger points to improve range of motion, reduce muscle stiffness tone and pain, increase muscle hyperemia, improve motor system mobility, and promote recovery, making it a valuable adjunct to sports rehabilitation and performance optimization programs [15]. An example of using the “encircle the dragon” method using dry needling is shown in Figure 2.

A. Ishak et al.’s study evaluated the impact of manual and laser ACU on the healing of burns in rats. The application of both methods resulted in significant improvements in macroscopic and microscopic healing parameters compared to the control group. Although there was no difference between ACU and laser ACU therapy, the results suggest the potential use of both methods as adjunctive therapies to accelerate burn healing [17].

As part of the experiment on the impact of ACU in the treatment of burns in rats, two groups with different observation periods, one hour or seven days, were formed. ACU was applied during bandage changes every other day. Pain and distress registration were conducted daily. The analysis showed that ACU reduces the accumulation of interleukin-6 in burn wounds, decreases the level of beta-endorphin in the blood, and promotes angiogenesis in the acute phase of burn injury [18].

ACU induces mechanical stress in connective tissue, particularly in fibroblasts and the cytoskeleton. This leads to mechanotransduction in connective tissue and the release of biochemical compounds. Thus, ACU can induce adaptation to impulses in the tissue environment and transmit information about the stimulus through sensory afferents [19].

During a systematic analysis of studies on acupuncture and DN treatment, it was found that the majority of research using needling techniques demonstrated a reduction in pain and other symptoms associated with scars [20]. According to A. Michalska et al., scar treatment should be comprehensive, involving physiotherapeutic methods, such as manual techniques, DN, cupping therapy, compression method, kinesiotaping, and physical interventions. These methods contribute to scar healing and prevention, being less invasive but economically accessible [21].

E. Rozenfeld et al. highlighted evidence that the application of the “dragon surrounding” method can alleviate pain in scars and wounds, as well as facilitate the wound healing process [16].

In recent years, we have observed great interest in DN and ACU therapies for treating scars, as posted on social media and in commercial courses. Analyzing the available contents, these therapies are not based on EBM (evidence-based medicine), and no confirmed, repeatable methodologies exist for conducting these therapies. This article analyzes the impacts of ACU and DN on the state of scars in the context of previous research, summarizes the results regarding the effectiveness of these methods, and determines the credibility of these data.

The aim of this study was to determine the effectiveness of DN and ACU therapies in the treatment of scar tissue and associated symptoms.

## 2. Materials and Methods

### 2.1. Registration

This systematic review adhered to the PRISMA 2020 recommendations, including the extension for review protocols (PRISMA-ScR) [22]. The systematic review’s predetermined plan was officially recorded on the Open Science Framework (OSF) platform, identified by the following registration number: osf.io/h3yeq.

### 2.2. Eligibility Criteria

The inclusion criteria for the study were defined according to the PICO methodology following the Cochrane principles [23].

Participants (P): The study included articles related to the treatment of patients of different ages and genders with colloid or hypertrophic postoperative scars of various origins.

Intervention (I): The intervention types included local treatment of scar tissue with dry needling or acupuncture and a combination of these treatment methods. Studies investigating the treatment of DN and ACU scars in combination with other physiotherapy methods were not excluded.

Comparators (C): Comparisons were made with a control group that did not receive treatment, received a sham treatment, or was treated with other physiotherapeutic methods.

Outcomes (O): The analysis considered changes in pain perception, range of motion, and itching related to the scar, as well as local transformations in scar characteristics. Psychological, functional, and overall physical patient statuses were taken into account, along with subjective assessments of the effectiveness of scar treatment.

Study Design (S): Preference was given to RCTs (randomized controlled trials) and studies with a control group. The inclusion criteria for the publication year covered 2020–2024, and studies from any country and author were considered. During the literature search, the inclusion criteria for year and study type were expanded to obtain a sufficient number of publications. The literature was reviewed up to the date of 16 January 2024, including case reports and case series. Articles with closed access, unpublished results, and those related to scar treatment from acne and microneedling in this context were excluded.

### 2.3. Information Sources

This study conducted a systematic search for scientific information on the impact of acupuncture and dry needling in scar tissue treatment using the PubMed, Cochrane Library, Semantic Scholar, Europe PubMed Central, and Google Scholar databases until 16 January 2024.

### 2.4. Search Strategy

The logical operators AND/OR were used to search for relevant literature. The code used for these searches is represented by the following specific string: “scar*” [Title/Abstract] AND (“acupuncture*” [Title/Abstract] OR “dry needle*” [Title/Abstract]). The search in the Semantic Scholar database was performed using the following keywords: “scar”, “acupuncture”, and “dry needling.”

### 2.5. Selection Process

**Figure 3:**
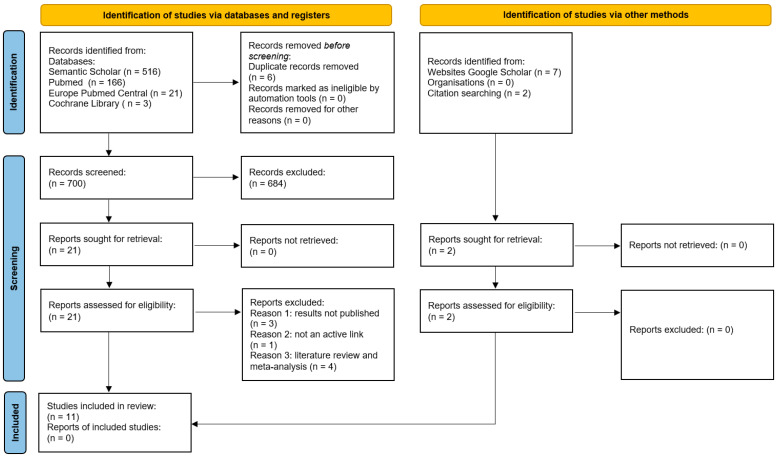
The PRISMA flowchart for the selection of the literature meeting the study’s criteria, according to M. Page et al. [22].

The selection of the relevant literature was carried out using the PRISMA flowchart. The titles of the search results were manually reviewed by two independent reviewers, and relevant studies underwent analysis of the main text. Through manual searching on Google Scholar, an additional 7 results were obtained, with 3 of them being duplicates from previous searches (Figure 3). Reviewing the list of article references led to the discovery of two more results (Anderson, 2014 [24]; Huang, 2020 [25]). After analyzing the titles and full texts of the articles, it was determined that 19 of them were relevant to the research topic. Three studies lacked results (Pourahmadi, 2023 [26]; Liu, 2020 [27]; Dale, 2017 [28]), and one reference to a study was found to be inactive (Hunter, 2011 [29]).

Articles that underwent a full review were structured using Microsoft Excel 2013 tables.

### 2.6. Data Collection Process

To organize, compare, and simplify the presentation of information, found articles on the topic and data extracted from each study were organized into a table using Microsoft Excel 2013 electronic spreadsheet software. The data compilation was conducted by two authors, each working independently. This structured data form allowed for the organization, comparison, and simplification of information.

### 2.7. Risk of Bias Assessment

The quality assessment of the studies was conducted using the JBI critical appraisal checklist for case reports and case series. For the quality assessment of the RCTs, the Physiotherapy Evidence Database (PEDro) scale was used. The quality assessments were performed by two authors, and the data were compared to derive a final assessment for each study.

### 2.8. Data Elements

Data extracted from the selected studies encompassed the authors’ last names, publication year, study type, and objectives. Details regarding the intervention covered the treatment duration, frequency, and needle type and size, as well as insertion angle. Scar-related information pre- and post-treatment, such as elasticity, density, color, sensitivity, and blood supply, along with evaluations of pain progression, itching, joint mobility, and physical functionality, was also gathered.

### 2.9. Data Synthesis Methods

The chosen research strategy was a systematic review, as it was not possible to unify the information because of the substantial variations in the methodologies and study results. The analysis of the results was conducted by addressing the critical question relevant to the topic, as follows: can the therapeutic effect of needling on scar tissue and associated symptoms be refuted or confirmed?

## 3. Results

During the literature search in electronic databases, 706 results were found. Table 1 shows the basic characteristics of the articles included in this systematic review.

The quality assessment of the case reports is presented in Table 2. All reports pertain to cases with a clear focus on demographic characteristics, patient histories, and clinical conditions. The majority of reports (seven out of eight) provide a clear description of the diagnostic tests and results. Six out of eight reports clearly describe interventions or treatment procedures. Adverse events or harm are clearly defined and described in only three out of eight reports (Tuckey, 2022 [3]; Huang, 2020 [25]; Bintoro, 2022 [33]). Most case reports fall within the range of 6–8 points, indicating more comprehensive reporting according to the respective criteria and an above-average study quality.

The series of cases studies (Lubczyńska, 2023 [30]) met the criteria related to the clear reporting of clinical information, results, and appropriate statistical analysis, receiving moderately high ratings (Table 3). However, it fell short in areas such as sequential participant inclusion, comprehensive participant inclusion, clear reporting of demographic indicators, and presentation of demographic information for the site(s)/clinic(s).

The methodological quality in terms of the research design and reporting in the RCTs, assessed using the PEDro scale, was rated as good (Kotani, 2001 [32]) and satisfactory (Song, 2011 [36]) (Table 4).

Each study had its unique purpose, such as evaluating the effectiveness of ACU in reducing pain, itching, restoring functionality, and treating symptoms (Table 5). Various types of scars were treated, including mature scars, hypertrophic burn scars, surgical scars, keloid scars, scars after laparotomy, and others.

All studies, except one (Tuck, 2010 [37]), report certain positive shifts in the treatment of patients with scars (Table 6). After treatment, improvements were observed in muscle strength and physical functionality. Physical activity tests, such as the “up and go” and the “30 s chair stand test”, showed enhancements in the results. The SF-36 indicated improvements in various aspects of patients’ quality of life. Physical functioning increased from 40% to 65%, indicating an improvement in the patient’s physical condition [2]. After treatment, the POSAS significantly decreased to 27/70 (38%), indicating a significant improvement in a scar’s appearance [3]. Visible improvements were noted in the VSS, particularly for color, vascular distribution, thickness, and scar flexibility [25].

## 4. Discussion

Modern scar treatment methods include corticosteroids (strips/patches or injections), chemotherapeutic agents, surgical excision, and radiation therapy. Conservative measures include compression therapy, gels, laser therapy, cryotherapy, and massages [7].

ACU on scar tissue is a procedure whose impact is difficult to assess unequivocally. An analysis of the research results revealed the use of different types of needles (0.15 mm × 15 mm, 0.30 mm × 50 mm, and 0.20 mm × 40 mm) and various techniques for their insertion (at an angle, parallel, or perpendicular). Most methodologies involve regular treatment sessions, which may be conducted 2–3 times per week, with treatment durations ranging from 3 to 8 weeks. Session times varied from 5 min to 1 h. Some studies combined ACU with other methods, such as infrared radiation, ultrasound, taping, and manual therapy, and in some treatment groups, diclofenac and Mebo Scareducer ointment were used, complicating the analysis of the specific impact of ACU.

In a recently published systematic review, the authors highlight the lack of scientific evidence, particularly RCTs, demonstrating the possible effectiveness of needling in cases of scar thickness, redness, elasticity, or limited range of motion [20]. The authors indicated that a meta-analysis was impossible because only two randomized trials and eight case reports were eligible for review; scar rating scales and pain severity scales were highly heterogeneous. The authors rightly state that the most numerous case studies on this topic are at high risk of bias and cannot determine the effectiveness of these forms of therapy [20].

Studies were conducted by different authors in different years, contributing to a diversity of perspectives and contexts. The case report results are not sufficient grounds for conclusions about treatment effectiveness, as they can be highly subjective and not generalizable to the entire population. Every scar is unique, even the routine chirurgical ones exhibit differences among patients. Furthermore, the healing process is affected by many individual factors. These facts lead to the conclusion that it is extremely difficult to gather together a large group with a high level of heterogeneity. Still, comparing the case studies to the randomized controlled trials and objective case series carries more scientific weight in establishing the effectiveness of treatment methods.

The results of the scar assessments before and after acupuncture and dry needling indicate significant improvements in muscle strength and functional tests, enhancement of the quality of life, and emotional well-being. There was noticeable relief in scar conditions, restoration of normal skin color, and a 50% reduction in pain, accompanied by the restoration of normal skin warmth.

Some visual improvements and subjective patient feelings observed as a result of ACU treatment [24] may undergo changes over time, and these changes are not always directly related to the intervention itself. Within 6–8 weeks after injury, hyperemia and densification of scar tissue are observed. Over the months, they may start to fade, stretch, and soften [7]. Such a process is characteristic of natural tissue healing and can influence the final outcome of ACU.

The reduction in pain, measured using the VAS and NRS, indicates a certain level of effectiveness of ACU and dry needling in reducing scar pain. In particular, observations show a significant decrease in pain from 8/10 to 3/10 on the VAS scale [2] and from 7/10 to 4.5/10 on the NRS scale [3]. Indicators 7 months post injury also suggest a certain level of sustained effect, although the reduction in pain was lower compared to after the initial treatment phase [3]. The control group did not show a reduction in pain, emphasizing the effectiveness of intradermal needling itself [32]. Therefore, the results suggest a certain potential of ACU and dry needling in treating scar pain.

According to some studies, ACU can inhibit inflammatory factors, provoke the synthesis of prostaglandin-E2 and nitric oxide, and stimulate the release of antioxidants [38]. ACU therapy can inhibit vascular endothelial growth factor (VEGF) in hypertrophic scars [39]. Uncontrolled VEGF is known to impede scar tissue formation during wound healing [40].

In the treatment of scar tissue with dry needling, the needle’s rotation stimulates fibroblast activity, promoting collagen release and faster tissue restoration; that is, winding the connective tissue around the inserted needle leads to the further displacement of the surrounding cellular matrix. Cellular remodeling plays a key role in reducing inflammation and pain [41]. The transmission of signals to the cells of connective tissue or the stimulation of sensory nerve fibers leads to the release of biologically active substances in the acupuncture area. Different needle movement methods were used in the studies, including a rocking motion [25] and a rapid rotational movement back and forth [2,34].

According to B.G. Wang et al., the ACU effect depends on the needle’s movements. Rotational movements contribute to greater analgesia, release of neurotransmitters, and more effective inflammation reduction [42]. The lift–thrust method can change the morphological structure of the thickened connective tissue and muscle layer; the input energy of this method is greater, especially with an increased needle diameter. Thus, the lift–thrust method may provide greater stimulation compared to rotation.

The sensation of itching arises because of the compression of nerves in scar tissue during wound healing, and intensified stimulation of nociceptors leads to painful sensations [43]. A decrease in itching from NRS = 5/10 to NRS = 4/10 may indicate some positive effects of the treatment, although this reduction was not very significant [3].

There is information about the impact of ACU on immunomodulation via applying pressure to connective tissue, stimulating hormonal changes, and influencing the central nervous system [44]. There is no consensus on this topic in the scientific community. J. Dunning et al. claim that the needle tip touches, taps, or pierces nociceptors [10]. However, some authors argue that nerve tissue, not connective tissue, is involved in ACU’s effect [45]. It was demonstrated that manually twisting ACU needles effectively suppressed artificially induced pathological conditions in rats. Importantly, these ACU effects were annulled using the local anesthetic bupivacaine, specifically by blocking the afferent nerve, indicating the crucial role of nerve tissue in generating the effects. Interestingly, disrupting connective tissue with type I collagenase at ACU points did not impact the ACU effects. This underscores the fact that, although connective tissue is present at acupuncture points, its contribution to these effects may be insignificant; although, according to H.M. Langevin et al., 80% of ACU points are located in connective tissue [46].

Some scar assessment results before and after acupuncture and dry needling indicate improved muscle strength and functional tests, quality of life, and emotional well-being. However, the authors did not provide information on the impact mechanisms and did not rule out the placebo effect. The improvements in well-being still need more reliable assessments. There were no results on muscle stiffness, elasticity, and tension using advanced, repeatable measurement techniques, such as tensiomyography, myotonometry, electromyography, or elastography.

The assessment of the effectiveness of the methodological approaches to ACU and DN in treating scars revealed that there are significant limitations commonly found in the studies included in this systematic review relating to the reporting of doses, specific treatment procedures, and intervention details. Still few studies followed the Standards for Reporting Interventions in Clinical Trials of Acupuncture (STRICTA) guidelines [47]. This observation is consistent with a previous review by Chmielewska et al. [20], who highlighted inconsistencies in the reporting of dry-needling dosing parameters and adverse events. Failure to accurately determine the impact of dry-needling doses on outcomes may introduce bias in decisions regarding its clinical effectiveness or optimal dosing. The notion of “dose” is neither clearly defined in the scientific literature nor in the education materials of practitioners, which leads to inconsistences. Regarding “dose”, for the purpose of this study, we considered all parameters related to the pattern of the needle’s application, number of needles, duration of the intervention, dimensions of the needles, additional stimulation (e.g., electrical), additional interventions (e.g., herbs), and application technique, as described in the STRICTA guidelines (see URL accessed on 1 July 2024 https://stricta.info/).

Future research should prioritize improving the reporting of treatment procedures, doses, and side effects by adopting standard reporting protocols tailored to these specific elements. Furthermore, in terms of evidence-gap mapping, there is a need to include a wider range of neuromuscular and physiological outcomes in experimental studies. Additionally, longitudinal cohorts are recommended to provide a comprehensive, long-term perspective on the effects of DN and ACU in scar management

In general, scars can lead to various negative effects, such as pain, itching, limited mobility, and social discomfort. In some clinical cases, scar tissue may pose challenges in its treatment. Studies aimed at evaluating the effectiveness and impact of acupuncture and dry needling in the treatment of different types of scars, such as postscar neuralgia, degenerative lower back pain, neuropathic scar pain, and hypertrophic scars, were analyzed. This systematic review shows that the use of acupuncture and dry-needling treatments can lead to successful outcomes, taking into account the improvements in physical conditions, reductions in pain, and positive effects on the patients’ qualities of life.

## 5. Limitations

In addition to the limitations that we elaborated on in discussion, there are more which have to be highlighted. The main limitation of this review is the low number of studies that could be included. In addition, the inability to directly compare the studies, as is performed in meta-analyses, because of the differences in the methodologies used by the different researchers is also a limitation. Because many of the included articles are case studies, which by definition describe a single case, the number of cases included in this review was low.

## 6. Conclusions

Each scientific study contributes to our understanding of the effectiveness of a given scar treatment method. Most studies included in this review were of moderate to high quality. Using various testing and assessment tools, improvements in the visual characteristics of scars, mobility, and overall physical and emotional well-being of the patients were observed, as well as pain relief and a reduction in itching. In terms of methodological reporting, there needs to be more consistency in the reporting of adverse events, particularly in the details of dry-needling procedures and doses, which are crucial for ensuring reproducibility. The most frequently studied result is the perception of pain and tissue mobility. At the same time, the functioning of the musculoskeletal system (including muscle strength and biomechanical properties, such as flexibility and stiffness) has rarely been examined, and it is characterized by a greater variety of reporting methods.

On the basis of the data provided, we conclude that ACU and dry-needling treatment may improve the physical conditions and quality of life of patients with scars. However, the limited sample of patients, the minimal number of studies, and the lack of evidence and hypotheses on the physiological mechanisms of the impact of DN and ACU on scars do not allow for a clear statement regarding the effectiveness of needling in the treatment of scars. The diversity of existing research results makes it difficult to generalize, conduct systematic analysis, and formulate clear conclusions. The guidelines and protocols for scar treatment have significant gaps, which are particularly important for practical implications. The next steps include ongoing monitoring and research to confirm the long-term effectiveness and safety of ACU and dry-needling treatment in patients with scarring.

## 7. Supporting Data

The materials used for this review include the PRISMA 2020 checklist for systematic reviews, the “PICO” framework for review assessments, and the PRISMA 2020 flow diagram for new systematic reviews, which involved searches in databases, registers, and other sources, as well as the JBI tools for the critical appraisal of systematic reviews, case reports, and case series. The sources can be found through the respective references.

## Figures and Tables

**Table 1:** Selection of studies.

	Name of the Author and Year	DN	ACU	ACU and DN or Else
1	Bahramian, 2022 [2]	case report		
2	Chmielewska, 2024 [20]			meta-analysis
3	Rozenfeld, 2020 [16]	literature review		
4	Dunning, 2014 [10]	literature review		
5	Pourahmadi, 2023 [26]	results not published		
6	Lubczyńska, 2023 [30]	case series		
7	Tuckey, 2019 [31]		literature review	
8	Tuckey, 2022 [3]		case report	
9	Kotani, 2001 [32]		RCT	
10	Huang, 2020 [25]			Fu’s subcutaneous needling, case report
11	Bintoro, 2022 [33]		case report	
12	Fang, 2014 [34]		case report	
13	Liu, 2020 [27]	results not published		
14	Khan, 2019 [35]	case report		
15	Song, 2011 [36]		RCT	
16	Dale, 2017 [28]			no results
17	Tuck, 2010 [37]		case report	
18	Anderson, 2014 [24]		case report	
19	Hunter, 2011 [29]		not an active link	

**Table 2:** JBI Critical Appraisal tools applied to the case reports on the use of ACU and DN in the treatment of scars.

Name of the Author and Year	A	B	C	D	E	F	G	H	ST
Bahramian, 2022 [2]	1	1	1	1	1	1	0	1	7
Huang, 2020 [25]	1	1	1	1	0	1	1	1	7
Bintoro, 2022 [33]	1	1	1	1	1	0	1	1	7
Fang, 2014 [34]	1	0	1	1	1	0	0	1	5
Khan, 2019 [35]	1	1	1	1	0	0	0	0	4
Tuck, 2010 [37]	1	1	1	1	1	1	0	1	7
Anderson, 2014 [24]	1	1	1	1	0	1	0	1	6
Tuckey, 2022 [3]	1	1	1	1	1	1	1	1	8

Note: ST—score total, 0–10 points, according to the Critical Appraisal tools, 2020, created by the author. A—Were the patient’s demographic details adequately outlined? B—Was the patient’s history carefully outlined and presented as a timeline? C—Was the patient’s current clinical status upon presentation explicitly detailed? D—Were diagnostic tests or assessment methods and the results clearly described? E—Was the intervention(s) or treatment procedure(s) clearly described? F—Was the postintervention clinical condition clearly described? G—Were adverse events or unanticipated events identified and described? H—Does the case report provide takeaway lessons?

**Table 3:** JBI Critical Appraisal tools applied to the case series on the use of ACU and DN in the treatment of scars.

Name of the Author and Year	A	B	C	D	E	F	G	H	I	J	ST
Lubczyńska, 2023 [30]	1	1	1	0	0	0	1	1	0	1	6

ST—score total, 0–10 points, according to the Critical Appraisal tools, 2020, created by the author. A—Were there clear criteria for inclusion in the case series? B—Was the condition assessed using a standardized and reliable method for all participants in the case series? C—Were valid methods used for identification of the condition for all participants included in the case series? D—Did the case series perform the consecutive inclusion of participants? E—Did the case series have complete inclusion of participants? F—Was there clear reporting of the demographics of the participants in the study? G—Was there clear reporting of clinical details of the participants? H—Were the outcomes or follow-up results of cases clearly reported? I—Was there clear reporting of the presenting site(s)’s/clinic(s)’s demographic information? J—Was the statistical analysis appropriate?

**Table 4:** Evaluation of the quality of the RCTs in scar treatment with ACU and DN according to the PEDro scale.

Name of the Author and Year	A	B	C	D	E	F	G	H	I	J	K	ST
Kotani, 2001 [32]	1	1	1	1	0	0	1	1	1	1	1	8
Song, 2011 [36]	1	1	0	1	0	0	0	1	1	1	0	5

ST—score total, 0–10 points, according to the PEDro scale, 2023, created by the author. A—Eligibility criteria were specified; B—subjects were randomly allocated to groups; C—allocation was concealed; D—groups were similar at baseline regarding the most important prognostic indicators; E—all subjects were blinded; F—all therapists who administered the therapy were blinded; G—all assessors were blinded who measured at least one key outcome; H—data on a specific outcome were obtained from over 85% of the subjects; I—all participants received the treatment or control condition as defined; J—the study presents statistical comparisons between groups for at least one significant outcome; K—the study provides both point measures and measures of variability for at least one key outcome.

**Table 5:** Data on the research of treatments with ACU and DN—material and methods.

Name of the Author, Year, Type of Research	Type of Scar	Study Objectives	Intervention
Bahramian, 2022 [2], case reports	Mature scar after hip joint surgery, longitudinal scar on the right measuring 51 × 4.8 cm, pink, firm, very sensitive to touch; on the left—38 × 3.6 cm, 2 months	Assessment of the effectiveness of DN in the treatment of scars regarding pain, range of motion, and functionality after hip joint replacement.	The needle 0.30 × 50 mm was inserted into the scar area, needles sized 0.25 × 25 mm were positioned above the proximal border of the scar strip at angles of 15° to 30° perpendicular to the surface of the scar. The needle was moved back and forth 2–3 times per session, 2 times/week for 3 weeks, for 30 min. Additionally, infrared radiation was applied for 20 min at a distance of 40 cm away from the body.
Tuckey, 2022 [3], case reports	Hypertrophic burn scar (pain, itching), skin graft on the left lateral surface of the chest, 3 months	Evaluation of clinical outcomes using local ACU for treating scar symptoms in a patient who has already recovered from a burn injury.	5 min of scar massage and 15 min of ACU, 12 sessions over 7 weeks. A maximum of 20 needles, each 2 cm around the scar, were inserted subcutaneously to a depth of 10 mm at a 45° angle, with the 20 mm needle shaft inserted at an angle under the edge of the scar. A maximum of 20 needles, each 2 cm around the scar, were inserted subcutaneously to a depth of 10 mm around the scar, positioned at a 45° angle. Additionally, a needle shaft measuring 20 mm was inserted beneath the edge of the scar at an angle.
Huang, 2020 [25], case reports	Curved surgical incision, 15 cm in length, with significant scarring on the neck, 8 years	Report a case of subcutaneous adhesions and scar tissue on the neck.	The doctor selected the “taut muscle” and inserted a needle into the taut subcutaneous tissue, either parallel or perpendicular to the muscle fibers. The needle was secured, and a “rocking motion” was performed for 2 min at a 40° angle, during which the patient actively or passively contracted the affected muscle. Treatment was administered 2–3 times per week for 1 month.
Bintoro, 2022 [33], case reports	Transverse dark-brown firm scar at the midlevel between the xiphoid process and the navel, measuring 30 × 0.3 cm, following laparoscopy, 4 years	Evaluate the effectiveness of the combination of BFA and ACU for treating scar pain after laparotomy.	ACU using BFA points on the ears, additional needles 0.15 × 15 mm were placed along the scar at a depth of 1–2 mm without further manual or electrical stimulation; for 12 sessions over 8 weeks, 30 min each.
Fang, 2014 [34], case reports	Surgical scar at the upper part of the right thigh along the gallbladder channel, 3 inches in length, 1/4 inch in width, red in color, and firm and hard, 1 year	Demonstrate the analgesic properties of ACU in scars.	Needles were used for insertion into local points: Wei Ci (8 needles at a 45° angle into the scar area), Hegu-LI-4, Taichong-LIV-3 (perpendicular to a depth of half cun, without De Qi sensation), and Zusanli-ST-36 (perpendicular to a depth of 1 cun, rapidly turning back and forth), 2/3 weeks, then 1/week for 2 weeks. Needles were single-use and remained in the body for 20 min for each procedure; 8 sessions over 5 weeks, 0.20 mm × 40 mm.
Khan, 2019 [35], case reports	Scar on the left thigh, 8 years	Present the treatment of DN for postscar neuralgia.	8 sessions of 1 h each, needles inserted into the scar tissue and around it.
Tuck, 2010 [37], case reports	Scar after mastectomy, 4 months	Assessing the effectiveness of acupuncture in managing lower back pain and neuropathic scar pain.	6 needles, with sizes of 0.16 × 30 mm, inserted perpendicular to the skin at a depth of 0.5 mm, 1–2 inches away from the scar line after mastectomy, using the “dragon surrounding” technique, for 30 min. Morphine per os 1 session.
Anderson, 2014 [24], case reports	Postoperative keloid scar on the palm of the right hand, 5 weeks	Present the impact of ACU on the postoperative scar.	Support points are located extrasegmentally. Over 3 months, 7 procedures involved inserting needles into the scar tissue for 20 min each. With ACU, ultrasound, stretching exercises, and splinting were applied. Needle sizes used: 0.20 × 40 mm, 0.3 × 50 mm.
Kotani, 2001 [32], RCT	Surgical scar ≥ 12 weeks	Testing the hypothesis that the introduction of intradermal needles reduces pain in scar tissue.	In the main group (n = 23), needles were inserted into painful areas, as determined using a pressure device (pain ≤ 2.5 kg/cm^2^). In the sham treatment group (n = 23), needles were inserted into nonpainful points, and in the control group (n = 24), no needles were inserted. Needles (0.16 mm × 5 mm) were left in throughout the day for a total of 20 sessions over 4 weeks. Diclofenac was taken as needed.
Song, 2011 [36], RCT	Hypertrophic scars on the abdomen, chest, limbs, and face, from 3 months to 4 years	To observe the outcomes of acupuncture therapy on scars.	Main group (n = 40) ACU + ultrasound therapy, control group (n = 40) Mebo Scareducer ointment + ultrasound therapy. Needles: 0.30 × 40–60 mm, inserted subcutaneously near the scars/around them; needles were manipulated 2–3 times and held for 30 min.
Lubczyńska, 2023 [30], case series	Postoperative scars on the elbow joint (1), abdominal cavity surgeries (3), and C-sections (7). Average age of 5 months (±2.9)	Assess the effectiveness of manual therapy in combination with other methods for treating postoperative scars.	n = 11, manual scar manipulation, massage, mobilization, DN (2 sessions/entire protocol, “dragon wrapping”), taping, 30 min each for 8 weeks, “dragon wrapping”.

BFA (battlefield acupuncture).

**Table 6:** Data on the treatment research of ACU and DN—effects.

Name of the Author, Year, Type of Research	Pain Assessment	Itching Assessment	Scar Assessment	
			Before Treatment	After Treatment
Bahramian, 2022 [2], case reports	VAS 8/10 before, 3/10 after	Not conducted	MMT: Flexion Rt. (Right): 4/5—Lt. (Left): 4+/5, Extension Rt.: 4/5—Lt.: 4−/5, Abduction Rt.: 4−/5—Lt.: 4/5, Adduction Rt.: 4−/5—Lt.: 4/5, External Rotation Rt.: 4/5—Lt.: 4−/5, Internal Rotation Rt.: 4/5—Lt.: 4−/5. Up and go test time: 7.49 s; 30 s chair stand test: 8 repetitions; SF-36 physical capabilities = 40%; psychological wellness 44%; social functioning 37.5%.	MMT: flexion Rt. (Right): 5−/5—Lt. (Left): 5−/5, extension Rt.: 5/5—Lt.: 5−/5, abduction Rt.: 4+/5—Lt.: 4+/5, adduction Rt.: 5/5—Lt.: 5−/5, external rotation Rt.: 5/5—Lt.: 5/5, internal rotation Rt.: 5/5—Lt.: 5/5. Up and go test time: 5.19 s; 30 s chair stand test: 12 repetitions; SF-36 physical capabilities = 65%; psychological wellness 56%; social functioning 62.5%.
Tuckey, 2022 [3], case reports	NRS = 7/10 before, NRS = 4.5/10; seven months after the injury NRS = 6/10	NRS = 5/10 before, NRS = 4/10 after	POSAS = 57/70 (81%). Summary scores (%): PCS—29, MCS—46. Domain scores (%): PF—15 (74), RP—44 (71), BP—31 (75), GH—45 (81), VT—44 (60), SF—63 (91), RE—38 (88), MH—75 (87).	POSAS = 27/70 (38%). After 7 weeks of treatment, the patient declined to complete treatment. After 7 months postinjury, it was not possible to complete over the phone.
Huang, 2020 [25], case reports	Not conducted.	Not conducted	VSS: M1, V0, H2, and P4, resulting in a total score of 7 points. The extent of neck movements in every directions was as outlined below: flexion: 30.67° ± 7.87°; extension: 38.83° ± 7.25°; right lateral flexion: 27.83° ± 3.66°; left lateral flexion: 26.00° ± 2.97°; right rotation: 54.83° ± 9.09°; left rotation: 53.67° ± 10.82°.	VSS was M1, V0, H2, and P2, resulting in a total score of 5 points. The extent of neck movements in all directions was as outlined below: Flexion: 38.83° ± 3.82°; extension: 41.83° ± 7.33°; right lateral flexion: 33.33° ± 2.50°; left lateral flexion: 28.33° ± 1.63°; right rotation: 58.33° ± 9.00°; left rotation: 62.00° ± 6.54°.
Bintoro, 2022 [33], case reports	NRS = 8/11	Not conducted	Transverse dark-brown firm scar at the midlevel between the xiphoid process and the navel, measuring 30 × 0.3 cm, following laparoscopy.	The scar is not described.
Fang, 2014 [34], case reports	7/10 on a Likert scale before, 1–2/10 after	Not conducted	Surgical scar in the upper part of the right thigh along the gallbladder channel, 3 inches in length, 1/4 inch in width, red in color, firm, and hard.	Less red than before the treatment.
Khan, 2019 [35], case reports	Not reported	Not reported	Not reported.	50% pain relief, in the patient’s words.
Tuck, 2010 [37], case reports	VAS 3–4/10 before, VAS 3–4/10 after	Not conducted	Not reported.	Not reported.
Anderson, 2014 [24], case reports	Not conducted	Not conducted	Cold and numbness in the pinky finger.	The finger became warm to the touch, and the skin color was normalized.
Kotani, 2001 [32], RCT	Continuous, sharp scar pain resistant to conventional treatment. After treatment—pain reduction by >70%, VAS = 0 in >40% of the treatment group; sham treatment group—pain reduction < 15%; no change in the control group	Not reported	Not reported.	Not reported.
Song, 2011 [36], RCT	Specifically not described	Specifically not described	The therapy assessment included treatment, outcomes, and failures. A 3-point scale was used to assess color, itching, and hardness, with severely scarred areas receiving 9 points (out of 80), moderate indications scoring 6–9 points (23 areas), and mild assessments scoring 1–5 points (15 areas).	Treatment criteria: in the treatment group—cure: n = 31; improvement: n = 15; failure: n = 3; in the control group—cure: n = 23; improvement: n = 12; failure: n = 10.
Lubczyńska, 2023 [30], case series	Following the treatment, a noteworthy disparity existed in the POSAS scores. Treatment had a significant positive influence on pain, pigmentation, pliability, pruritus, surface area, and scar stiffness. Improvement in skin parameters (scar elasticity, thickness, regularity, color) was also noticed	A noteworthy disparity existed in thePOSAS after treatment	Discomfort and pain in the scar area, hydration (37.8 ± 7.7), TEWL (g/m^2^/h) (13 ± 4), elasticity (mm) (0.003 ± 0.0003), and erythema level (352.1 ± 103.1). POSAS.	Hydration (48.6 ± 1.2), TEWL (9.7 ± 2.4), elasticity (0.05 ± 0.01), and erythema level (249.9 ± 89.8). Melanin level unchanged. POSAS shows a statistically significant difference.

Note: VAS (visual analogue scale), MMT (manual muscle testing), NRS (numerical rating scale for pain and itch), POSAS (Patient and Observer Scar Assessment Scale), VSS (Vancouver Scar Scale), M (color), V (vascularity), H (height), P (pliability), TEWL (transepidermal water loss), SF-36 (36-Item Short-Form Health Survey questionnaire).

## Data Availability

No new data were created for this study.
